# Correlation between structure/function and optic disc microcirculation in myopic glaucoma, measured with laser speckle flowgraphy

**DOI:** 10.1186/1471-2415-14-113

**Published:** 2014-09-24

**Authors:** Naoko Aizawa, Hiroshi Kunikata, Yukihiro Shiga, Yu Yokoyama, Kazuko Omodaka, Toru Nakazawa

**Affiliations:** Department of Ophthalmology, Tohoku University Graduate School of Medicine, 1-1 Seiryo-machi, Aoba-ku, Sendai, 980-8574 Japan; Department of Retinal Disease Control, Tohoku University Graduate School of Medicine, Sendai, Japan; Department of Advanced Ophthalmic Medicine, Tohoku University Graduate School of Medicine, Sendai, Japan

**Keywords:** Laser speckle flowgraphy, Glaucoma, Myopia, Myopic disc, Retinal nerve fiber layer, Optic nerve blood flow

## Abstract

**Background:**

It is difficult to identify glaucoma in myopic eyes because the configuration of the optic disc varies; yet it is important clinically. Here, we used laser speckle flowgraphy (LSFG) to measure mean blur rate (MBR), representing optic disc microcirculation, and assessed its ability to identify glaucoma in eyes with myopic optic discs.

**Methods:**

129 eyes (normal disc: 21 eyes; myopic disc: 108 eyes) were enrolled. The eyes were classified as normal or mildly, moderately, or severely glaucomatous with standard automated perimetry (SAP). We determined the relationship between optic nerve head (ONH) MBR, measured with LSFG, mean deviation (MD), measured with SAP, and circumpapillary retinal nerve fiber layer thickness (cpRNFLT), measured with optical coherence tomography (OCT).

**Results:**

ONH MBR and cpRNFLT decreased significantly with the severity of glaucoma. MBR was significantly correlated with cpRNFLT and MD (r =0.65 and r =0.63, respectively). A multiple regression analysis revealed that MBR and cpRNFLT were independent factors indicating glaucoma severity. A logistic regression analysis revealed that MBR and cpRNFLT were also independent factors indicating the presence of glaucoma. In a receiver operating characteristic (ROC) analysis, MBR and cpRNFLT could both differentiate between normal and glaucomatous eyes (MBR area under the ROC curve: 0.86, with a cut-off score of 24.0 AU).

**Conclusion:**

These results suggest that in addition to cpRNFLT, non-invasive and objective LSFG measurements of MBR may enable the identification of glaucoma and the classification of its severity in eyes with myopic optic discs.

## Background

The presence of a myopic optic disc makes it difficult to detect glaucoma, yet such a diagnosis is very important because myopia is such a common ophthalmological disorder, particularly in Asia and Oceania, and because glaucoma is the second most common cause of blindness worldwide [1–6]. The development of spectral domain optical coherence tomography (SD-OCT) has enabled ophthalmologists to quickly and easily measure the average thickness of the circumpapillary retinal nerve fiber layer (cpRNFLT). This has been reported in many studies to be a reliable way of detecting glaucoma, although standard automated perimetry (SAP) measurements of mean deviation (MD) of the visual field are still necessary, as they remain the standard method of diagnosing the disease. Glaucoma is characterized by the loss of retinal ganglion cells (RGCs) and their axons, which comprise the retinal nerve fiber layer (RNFL) [7], but glaucomatous thinning of cpRNFLT is difficult to measure in eyes with myopic discs because of various confounding factors, such as disc deformation, tilted disc and peripapillary atrophy [5, 8].

Recent innovations in laser speckle flowgraphy (LSFG) have allowed us to monitor changes in optic disc microcirculation, represented as mean blur rate (MBR), over time, at the same site and in the same eye. There have already been a limited number of reports that used MBR to evaluate blood flow [9–12]. Decreased MBR has also been reported to be associated with the severity of glaucoma in studies that included eyes with myopic glaucomatous discs [11, 12]. Though the pathogenesis of glaucoma in eyes with myopic optic discs remains unclear, as it does in normal tension glaucoma, decreased ocular blood flow [13], including optic nerve microcirculation, is considered to be a promising candidate, in addition to mechanical stress, oxidative stress and decreased axonal flow.

In this study, therefore, we hypothesized that glaucoma in eyes with myopic discs might be related to decreased microcirculation in the optic nerve head (ONH). To evaluate our hypothesis, we measured MBR in the ONH with LSFG and determined the correlation between MBR and the severity of visual field defects in eyes with myopic discs. We also determined the ability of LSFG examinations to identify glaucoma and classify its severity in these eyes. Thus, the purpose of this report was to evaluate the relationship between MD, cpRNFLT, and MBR in eyes with myopic discs.

## Methods

### Subjects

This case-control study comprised 108 eyes of 66 consecutive Japanese patients (M: F = 37: 29, mean age 54.3 ± 7.5 years) with myopic optic discs who visited Tohoku University Hospital in Miyagi, Japan from January 2009 to May 2010. Myopic optic discs were defined as being oval and temporally tilted with crescent peripapillary atrophy [6, 14]. Glaucomatous eyes were defined as those with glaucomatous optic neuropathy and a corresponding abnormal glaucomatous visual field according to the Anderson-Patella classification [15]. The criteria for an abnormal visual field included results outside the normal limits from a glaucoma hemifield test; the presence of a cluster of three or more non-edge points, all of which were depressed on the pattern deviation plot at a P < 5% level, with one depressed at a P < 1% level; and corrected pattern standard deviation with significance at a P < 5% level. SAP was performed with the Swedish interactive threshold algorithm (SITA)-standard strategy of the 30-2 program of the Humphrey field analyzer (HFA) (Carl Zeiss Meditec, Dublin, CA, USA). Reliability criteria for SAP included fixation errors < 20%, false-positives < 20%, and false-negatives < 20%. The eyes were classified into four groups according to the presence of glaucoma and the progression of glaucomatous visual field defects: normal, as well as mild (mean deviation: MD > -6.0 dB), moderate (MD -6.0 to -12.0 dB), and severe glaucoma (MD < -12.0 dB). Twenty-one eyes without myopic discs served as controls. Patients were excluded if they had ocular diseases other than open angle glaucoma or had systemic diseases affecting the visual field. The study was approved by the institutional review board of Tohoku University Graduate School of Medicine. Informed consent for the participation in the research was obtained from each patient and the research was conducted according to the provisions of the Declaration of Helsinki, 1995 (as revised in Edinburgh, 2000).

### Measurement of clinical parameters

All subjects underwent a funduscopic examination. MD was measured with HFA, cpRNFLT was measured with 3D OCT-2000 (version 8.00; Topcon, Inc., Tokyo, Japan), and IOP was measured with Goldmann applanation tonometry. All patients rested in a darkened room for 10 minutes before the start of the examination. Systolic and diastolic blood pressures (SBP and DBP) were recorded, following which three measurements of ONH circulation were made with LSFG. Averages of the measurements were used for the statistical analysis. The use of systemic medications for blood pressure control or topical anti-glaucoma treatment was also recorded.

### Mean blur rate in the optic nerve head

The pupils of each subject were dilated with 0.5% tropicamide and 0.5% phenylephrine hydrochloride. LSFG and blood pressure measurements were made after the subjects had rested for 10 minutes in a darkened room. In order to improve the consistency and reproducibility of the examinations, after each image was captured, the subjects briefly moved away from the device before repositioning themselves. The patient gazed at the fixation point of the device, at the center of their visual field, and the investigator was then able to control the eye’s position by moving the fixation point. The center of the captured image was set at a point mid-way between the optic disc and the macula. The position of the subject’s eye was saved by the capture software (LSFG Measure, version 6.64.00; Softcare Ltd., Fukutsu, Japan), enabling us to capture the same area in following examinations. When the image was not sufficiently focused, we instead used dark vertical lines on the live image. The parameters of MBR in optic disc used in this study were calculated by the equipped software (LSFG Analyzer, version 3.0.39.0, Softcare Ltd., Fukutsu, Japan). We determined the margin of the optic disc with a round rubber band. We then saved the position of each region of interest (ROI) in software, and reused it in subsequent analyses of the same patient.

### Statistical analysis

A one-way analysis of variance or a chi-square test were used to determine the significance of differences between the groups. The Mann-Whitney U test was used for the MBR and cpRNFLT data for the groups. Spearman’s rank correlation test was used to evaluate single correlations between variables (MBR and cpRNFLT, and MBR and MD). Multiple linear regression analysis was performed to determine the independent variables affecting glaucoma stage in eyes with myopic discs. Logistic regression analysis was performed to determine the independent variables contributing to glaucoma in eyes with myopic discs. The receiver operating characteristic curve (ROC) for MBR and cpRNFLT was plotted to determine the optimum cut-off point, and the area under the ROC curve (AUC) was calculated to determine the power of discrimination between eyes with normal visual fields and eyes with glaucoma. The ROC of MBR and cpRNFLT was compared statistically. These statistical analyses were performed with JMP software (Pro version 10.0.2, SAS Institute Japan Inc., Tokyo, Japan). The significance level was set at *P* < 0.05.

## Results

### Clinical characteristics

The analysis was based on 129 eyes (normal disc: 21 eyes; myopic disc: 108 eyes). Table 1 summarizes the characteristics of the eyes with normal and myopic optic discs. There were no significant differences in age, sex, SBP or DBP among any of the groups, including the normal eyes, but there were significant differences in IOP, spherical equivalent, MD, cpRNFLT and MBR. There were no significant differences in age, sex, SBP, DBP, hypertension medication, topical glaucoma medication or IOP among the myopic groups but there were significant differences in spherical equivalent, MD, cpRNFLT and MBR.

**Table 1:** **Characteristics of normal and myopic eyes with each stage of glaucoma**

Optic disc type	Normal	Myopic				*P*value	
Visual field severity		Normal	Mild	Moderate	Severe	All	Myopic
Number of eyes	21	28	39	18	23	-	-
Age (years)	57.7 ± 7.4	54.8 ± 6.2	54.5 ± 7.6	51.8 ± 9.1	55.3 ± 7.7	0.23^a^	0.48^a^
Sex (M:F)	12 : 9	17 : 11	22 : 17	13 : 5	13 : 10	0.82^b^	0.69^b^
SBP (mmHg)	123.911.8	128.0 ± 16.3	125.2 ± 14.5	126.7 ± 13.3	128.3 ± 19.6	0.84^a^	0.87^a^
DBP (mmHg)	74.0 ± 8.9	77.9 ± 12.4	75.9 ± 15.0	79.6 ± 15.1	81.2 ± 16.2	0.43^a^	0.56^a^
Hypertension medications (n, %)	-	2, 7.1	3, 7.7	0, 0	4, 17.3	-	0.10^b^
Glaucoma medications (n, %)	-	-	-	-	-	-	0.51^b^
Prostaglandin analogues	-	-	27, 69.2	14, 77.8	21, 91.3	-	-
Beta-anatagonists	-	-	10, 25.6	7, 38.9	13, 56.5	-	-
Carbonic anhydrase inhibitors	-	-	12, 30.8	5, 27.8	9, 39.1	-	-
Alpha-1-anatagonists	-	-	4, 10.3	2, 11.1	10, 43.5	-	-
IOP (mmHg)	13.0 ± 2.8	14.7 ± 2.8	17.0 ± 4.5	17.9 ± 6.3	17.4 ± 3.8	<.001^a^	0.05^a^
Spherical equivalent (D)	-1.5 ± 2.5	-3.1 ± 2.3	-4.4 ± 3.0	-6.0 ± 2.4	-5.0 ± 2.4	0.003^a^	0.003^a^
MD (dB)	0.8 ± 0.9	0.05 ± 1.1	-3.2 ± 1.8	-8.6 ± 1.2	-20.1 ± 3.7	<.001^a^	<.001^a^
CpRNFLT (μm)	118.3 ± 9.6	99.7 ± 11.6	84.3 ± 16.5	75.3 ± 15.0	61.3 ± 10.0	<.001^a^	<.001^a^
MBR (AU)	31.1 ± 4.0	26.3 ± 5.1	21.9 ± 5.4	18.2 ± 4.0	16.8 ± 4.6	<.001^a^	<.001^a^

AU = arbitrary units, cpRNFLT = circumpapillary retinal nerve fiber layer thickness,

D = diopter, DBP = diastolic blood pressure, IOP = intraocular pressure, MBR = mean blur rate,

MD = mean deviation, SBP = systolic blood pressure.

^a^One-way analysis of variance.

^b^Chi-square test.

### Relationship between MBR and other variables

**Figure 1:**
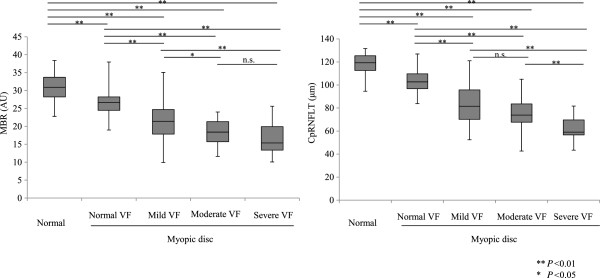
**Changes in MBR and cpRNFLT compared to disc type and severity of glaucomatous visual field progression.** MBR decreased significantly, except between the moderate and severe stages of visual field (VF) loss. CpRNFLT also decreased significantly with every stage of the disease, except between the mild and moderate stages of VF loss.

**Figure 2:**
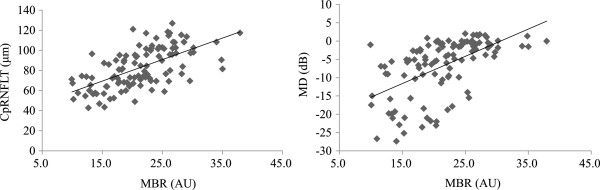
**Relationship between MBR and other clinical findings in eyes with myopic optic discs.** MBR was significantly correlated with cpRNFLT and MD (*P* < 0.001, r =0.65 and *P* < 0.001, r = 0.63, respectively, Spearman’s coefficient of correlation by rank).

ONH MBR and cpRNFLT were significantly lower depending on the presence and severity of glaucoma (Figure 1). MBR was significantly correlated with cpRNFLT and MD (*P* < 0.001, r =0.65 and *P* < 0.001, r =0.63, respectively, Figure 2). A multiple regression analysis revealed that MBR and cpRNFLT were independent factors indicating glaucoma severity (*P* =0.009 and *P* < 0.001, respectively, Table 2). A logistic regression analysis revealed that MBR and cpRNFLT were independent factors indicating the presence of glaucoma (*P* =0.02 and *P* < 0.001, respectively, Table 3).

**Table 2:** **Multiple regression analysis for factors independently contributing to glaucoma stage in eyes with myopic optic discs**

Variable			
Dependent	Independent	β	*P*value
Glaucoma stage	Age	-0.02	0.72
	IOP	0.05	0.50
	Spherical equivalent	-0.02	0.82
	CpRNFLT	-0.58	<.001
	MBR	-0.23	0.009

β = standard partial regression coefficient, IOP = intraocular pressure,

cpRNFLT = circumpapillary retinal nerve fiber layer thickness,

MBR = mean blur rate.

**Table 3:** **Logistic regression analysis of independently variables affecting the presence of glaucoma in eyes with myopic optic discs**

	Normal (N = 28)	Glaucoma (N = 80)	*P*value	Odds ratio (95% CI)
Age (years)	54.8 ± 6.2	54.2 ± 8.0	0.30	0.94 (0.84- 1.05)
IOP (mmHg)	14.7 ± 2.8	17.3 ± 4.3	0.02	1.26 (1.03- 1.65)
Spherical equivalent (D)	-3.1 ± 2.3	-4.9 ± 2.7	0.84	0.97 (0.73- 1.28)
CpRNFLT (μm)	104.0 ± 10.2	75.7 ± 17.4	<.001	0.91 (0.85- 0.95)
MBR (AU)	26.5 ± 3.9	19.8 ± 5.4	0.02	0.83 (0.70- 0.97)

IOP = intraocular pressure, cpRNFLT = circumpapillary retinal nerve fiber layer thickness,

D = diopter, MBR = mean blur rate, AU = arbitrary units.

### The ability of MBR and cpRNFLT to differentiate between normal and glaucomatous eyes with myopic optic discs

**Figure 3:**
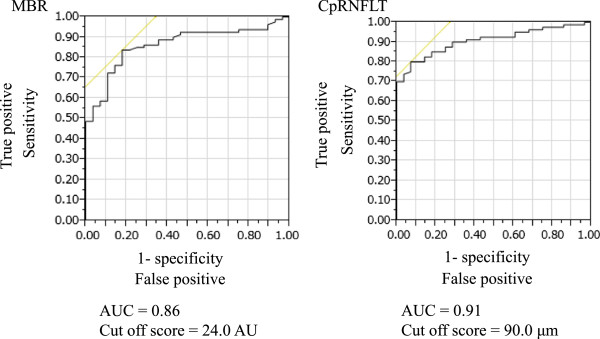
**Receiver operating characteristic curve for MBR and cpRNFLT in eyes with myopic optic discs.** Among the eyes with myopic optic discs, MBR and cpRNFLT could both differentiate between eyes with and without glaucoma (MBR AUC: 0.86, with a cut-off score of 24.0 AU; cpRNFLT AUC: 0.91, with a cut-off score of 90.0 μm), statistically similar results (*P* =0.25).

An ROC analysis (Figure 3) showed that both MBR and cpRNFLT could differentiate between normal eyes with myopic optic disc and glaucomatous ones (MBR AUC: 0.86, with a cut-off score of 24.0 AU; cpRNFLT AUC: 0.91, with a cut-off score of 90.0 μm). The differentiation power of MBR and cpRNFLT was statistically similar (*P* =0.25).

## Discussion and conclusion

We set out to evaluate the feasibility of using LSFG measurements of ONH MBR to diagnose glaucoma in myopic eyes. The myopic eyes with and without glaucoma in our study did not show any significant differences in clinical characteristics other than in IOP, spherical equivalent and cpRNFLT, but we found that reductions in the ONH MBR of these eyes were significantly associated with the severity of glaucoma. A multiple linear regression analysis showed that both ONH MBR and cpRNFLT were contributors to the stage of glaucoma severity. Furthermore, we found that ONH MBR and cpRNFLT were also independent factors indicating the presence of glaucoma in eyes with myopic discs. Finally, an ROC analysis revealed that the AUC for MBR could accurately predict the presence of glaucoma. Its accuracy was statistically similar to that of cpRNFLT.

It is well known that OCT measurements of cpRNFLT can identify the presence of glaucoma, and that cpRNFLT decreases with the severity of glaucoma [11, 16–20]. Our findings support these existing results, as well as results showing that MBR is correlated with MD and cpRNFLT in eyes with myopic discs [11]. Recent studies of ocular circulation suggest that the incidence and progression of glaucoma may be related to decreased perfusion [21–23]. Several large epidemiological studies have demonstrated that retinal arteriole and vessel narrowing is associated with a high incidence of glaucoma [24–27]. Interestingly, the Blue Mountains Eye Study also showed that retinal arteriole narrowing is associated with a long-term risk of OAG [27]. Furthermore, there have been many fluorescein angiography (FA) studies of eyes with glaucoma showing that the size of the filling defect is correlated with the severity of glaucoma [28–33]. However, FA examination is invasive, and the injection of fluorescein can cause severe complications, such as anaphylactic shock. The results can also be affected by time-dependent changes, making it difficult to quantify changes in ONH microcirculation clinically. There has thus been a need for a non-invasive method to measure ONH microcirculation. In response, a number of techniques using laser Doppler flowmetry have been used to demonstrate that ocular microcirculation decreases in glaucoma patients [34–36], and now, a newer technology, LSFG, has allowed us to measure ocular circulation quickly and non-invasively, as well as quantify microcirculation in the optic disc, choroid, and retinal vessels [37]. The main measurement parameter of LSFG is MBR, a relative index of the velocity of erythrocytes [9, 38], which has been reported to have high reproducibility in normal subjects as well as those with glaucoma [39]. Although MBR is a relative value, MBR and the results of MBR waveform analysis should be comparable between eyes [40], as suggested by recent studies that demonstrated the correlation of MBR with absolute ocular blood flow values measured with the microsphere or hydrogen gas clearance methods [41, 42].

Although OCT images of the cpRNFL can be a valuable source of indicators of glaucoma, the borderline of the disc is often unclear in myopic eyes because of the abnormal configuration of the disc. This makes automatic segmentation of the RNFL near the myopic disc prone to errors. In response, several studies have evaluated the diagnostic performance for glaucoma of measurements of macular ganglion cell complex (GCC) thickness, and have reported that its accuracy is similar to measurements of cpRNFLT, even in highly myopic eyes [43–45]. However, it has been demonstrated that the GCC is thin or absent in the fovea [43], and automatic segmentation of these very thin, unclear retinal layers in the macula is again prone to errors, leading to inaccurate diagnoses. The advantage of LSFG measurements of MBR is that they can be accurately made regardless of the configuration of the disc due to the high transparency of the ONH and the nature of LSFG. We believe this explains the strength of the correlation between MBR and glaucoma severity in eyes with myopic discs. MBR measurements with LSFG, despite their simplicity, should therefore be useful clinically and be considered a candidate biomarker of glaucoma in eyes with myopic discs.

Interestingly, our study is the first to show a significant difference between eyes with normal discs and non-glaucomatous eyes with myopic optic discs. We found that only eyes with myopic optic discs had decreased optic nerve microcirculation. However, although the MBR in myopic eyes without glaucomatous visual fields was similar to normal eyes, we speculate that preperimetric glaucoma (PPG) may have been present in some of these myopic eyes, and that there may be a cut-off value for MBR indicating progression to PPG. Another interesting finding was that the relationship between MBR and MD changed when MBR fell below 25 AU, although MBR and MD were correlated overall. (Figure 2, right). This indicates that the visual field was usually preserved with a mild decrease in optic disc microcirculation, but that a moderate or severe decrease could lead to either a severely impaired or relatively preserved visual field. Overall, however, MBR was closely correlated with cpRNFLT (Figure 2, left), indicating a close relationship between structure and microcirculation in eyes with myopic glaucoma. Thus, we believe that MBR undergoes decreases similar to those in cpRNFLT before visual field defects become apparent.

Our cross-sectional study had several limitations. It included only a small number of myopic eyes (approximately 100). Since the LSFG measurements were taken from glaucoma patients who had not discontinued the use of anti-glaucoma or hypertension medications, due to ethical considerations, we must note the possible impact of topical medications on MBR. Nevertheless, we did not find a significant difference in the number of topical glaucoma medications between the groups of patients with glaucoma. Regardless, the effects of topical anti-glaucoma medications on ONH circulation remain controversial. In particular, previous clinical studies reported that a number of topical prostaglandin analogues, as well as a combined topical therapy with a beta-antagonist and a carbonic anhydrase inhibitor, caused a significant increase in ONH circulation [46, 47]. On the other hand, there have also been studies that did not show that topical anti-glaucoma medications had any effect on ONH circulation [48, 49]. Topical anti-glaucoma medications also increase mean 24-hour diastolic ocular perfusion pressure (OPP), calculated from mean arterial pressure and IOP. The value of 24-hour OPP has been reported to be clinically relevant in glaucoma patients [50–52]. Additionally, eyes with myopic discs tend to have a range of IOP values, a phenomenon that could bias determinations of the correlation between structure/function and optic disc microcirculation. Furthermore, our results were obtained from only patients of Japanese ethnicity and cannot be directly applied to patients with other ethnic backgrounds, because of differing fundus pigmentation.

In conclusion, we found that LSFG measurements of MBR in the ONH were strongly correlated with MD and cpRNFLT in eyes with myopic optic discs. Furthermore, MBR was an excellent indicator of the presence of glaucoma in an ROC analysis, matching the accuracy of cpRNFLT. LSFG measurement of MBR may thus be useful as a non-invasive, objective evaluation of glaucoma in eyes with myopic optic discs. We believe that our data suggest that a simple MBR analysis of eyes with myopic discs may be of clinical use in identifying glaucoma and in making precise evaluations of its severity. Since optic nerve microcirculation in glaucomatous myopic eyes tends to decrease with the severity of glaucoma, replacing treatments aimed at reducing IOP with ones aimed at increasing optic nerve microcirculation may be of benefit to this category of glaucoma patients.

## Authors’ information

NA: MD, HK: MD PhD an Associate Professor, YS: MD, YY: MD, KO: MD, and TN: MD PhD a Professor of Department of Ophthalmology, Tohoku University Graduate School of Medicine, Sendai, Japan.

## Abbreviations

SD-OCT: Spectral domain optical coherence tomography
RGCs: Retinal ganglion cells
LSFG: Laser speckle flowgraphy
MBR: Mean blur rate
BCVA: Best-corrected visual acuity (BCVA)
FT: Foveal thickness
OCT: Optical coherence tomography
ONH: Optic nerve head
IOP: Intraocular pressure
LDF: Laser Doppler flowmetry
SAP: Standard automated perimetry
MD: Mean deviation
cpRNFLT: Circumpapillary retinal nerve fiber layer thickness
ROC: Receiver operating characteristic
AU: Arbitrary unit
SBP and DBP: Systolic and diastolic blood pressures
ROI: Region of interest
AUC: Area under the receiver operating characteristic
GCC: Ganglion cell complex
PPG: Preperimetiric glaucoma.

## References

[1] Quigley HA (1996). Number of people with glaucoma worldwide. Br J Ophthalmol.

[2] Resnikoff S, Pascolini D, Etya'ale D, Kocur I, Pararajasegaram R, Pokharel GP, Mariotti SP (2004). Global data on visual impairment in the year 2002. Bull World Health Organ.

[3] Mitchell P, Hourihan F, Sandbach J, Wang JJ (1999). The relationship between glaucoma and myopia: the Blue Mountains Eye Study. Ophthalmology.

[4] Xu L, Li Y, Wang S, Wang Y, Jonas JB (2007). Characteristics of highly myopic eyes: the Beijing Eye Study. Ophthalmology.

[5] Xu L, Wang Y, Wang S, Jonas JB (2007). High myopia and glaucoma susceptibility the Beijing Eye Study. Ophthalmology.

[6] Nakazawa T, Shimura M, Ryu M, Himori N, Nitta F, Omodaka K, Doi H, Yasui T, Fuse N, Nishida K (2012). Progression of visual field defects in eyes with different optic disc appearances in patients with normal tension glaucoma. J Glaucoma.

[7] Quigley HA, Dunkelberger GR, Green WR (1989). Retinal ganglion cell atrophy correlated with automated perimetry in human eyes with glaucoma. Am J Ophthalmol.

[8] Witmer MT, Margo CE, Drucker M (2010). Tilted optic disks. Surv Ophthalmol.

[9] Watanabe G, Fujii H, Kishi S (2008). Imaging of choroidal hemodynamics in eyes with polypoidal choroidal vasculopathy using laser speckle phenomenon. Jpn J Ophthalmol.

[10] Liang Y, Downs JC, Fortune B, Cull G, Cioffi GA, Wang L (2009). Impact of systemic blood pressure on the relationship between intraocular pressure and blood flow in the optic nerve head of nonhuman primates. Invest Ophthalmol Vis Sci.

[11] Yokoyama Y, Aizawa N, Chiba N, Omodaka K, Nakamura M, Otomo T, Yokokura S, Fuse N, Nakazawa T (2011). Significant correlations between optic nerve head microcirculation and visual field defects and nerve fiber layer loss in glaucoma patients with myopic glaucomatous disk. Clin Ophthalmol.

[12] Chiba N, Omodaka K, Yokoyama Y, Aizawa N, Tsuda S, Yasuda M, Otomo T, Yokokura S, Fuse N, Nakazawa T (2011). Association between optic nerve blood flow and objective examinations in glaucoma patients with generalized enlargement disc type. Clin Ophthalmol.

[13] Flammer J, Orgul S, Costa VP, Orzalesi N, Krieglstein GK, Serra LM, Renard JP, Stefansson E (2002). The impact of ocular blood flow in glaucoma. Prog Retin Eye Res.

[14] Nicolela MT, Walman BE, Buckley AR, Drance SM (1996). Various glaucomatous optic nerve appearances: a color Doppler imaging study of retrobulbar circulation. Ophthalmology.

[15] Anderson D, Patella V (1999). Automated Static Perimetry.

[16] Guedes V, Schuman JS, Hertzmark E, Wollstein G, Correnti A, Mancini R, Lederer D, Voskanian S, Velazquez L, Pakter HM, Pedut-Kloizman T, Fujimoto JG, Mattox C (2003). Optical coherence tomography measurement of macular and nerve fiber layer thickness in normal and glaucomatous human eyes. Ophthalmology.

[17] Wollstein G, Schuman JS, Price LL, Aydin A, Beaton SA, Stark PC, Fujimoto JG, Ishikawa H (2004). Optical coherence tomography (OCT) macular and peripapillary retinal nerve fiber layer measurements and automated visual fields. Am J Ophthalmol.

[18] Wollstein G, Ishikawa H, Wang J, Beaton SA, Schuman JS (2005). Comparison of three optical coherence tomography scanning areas for detection of glaucomatous damage. Am J Ophthalmol.

[19] Shoji T, Nagaoka Y, Sato H, Chihara E (2012). Impact of high myopia on the performance of SD-OCT parameters to detect glaucoma. Graefes Arch Clin Exp Ophthalmol.

[20] Akashi A, Kanamori A, Nakamura M, Fujihara M, Yamada Y, Negi A (2013). The ability of macular parameters and circumpapillary retinal nerve fiber layer by three SD-OCT instruments to diagnose highly myopic glaucoma. Invest Ophthalmol Vis Sci.

[21] Caprioli J, Coleman AL (2010). Blood pressure, perfusion pressure, and glaucoma. Am J Ophthalmol.

[22] Leske MC (2009). Ocular perfusion pressure and glaucoma: clinical trial and epidemiologic findings. Curr Opin Ophthalmol.

[23] Werne A, Harris A, Moore D, BenZion I, Siesky B (2008). The circadian variations in systemic blood pressure, ocular perfusion pressure, and ocular blood flow: risk factors for glaucoma?. Surv Ophthalmol.

[24] Amerasinghe N, Aung T, Cheung N, Fong CW, Wang JJ, Mitchell P, Saw SM, Wong TY (2008). Evidence of retinal vascular narrowing in glaucomatous eyes in an Asian population. Invest Ophthalmol Vis Sci.

[25] Wang S, Xu L, Wang Y, Wang Y, Jonas JB (2007). Retinal vessel diameter in normal and glaucomatous eyes: the Beijing eye study. Clin Exp Ophthalmol.

[26] Mitchell P, Leung H, Wang JJ, Rochtchina E, Lee AJ, Wong TY, Klein R (2005). Retinal vessel diameter and open-angle glaucoma: the Blue Mountains Eye Study. Ophthalmology.

[27] Kawasaki R, Wang JJ, Rochtchina E, Lee AJ, Wong TY, Mitchell P (2013). Retinal vessel caliber is associated with the 10-year incidence of glaucoma: the Blue Mountains Eye Study. Ophthalmology.

[28] Arend O, Remky A, Plange N, Kaup M, Schwartz B (2005). Fluorescein leakage of the optic disc in glaucomatous optic neuropathy. Graefes Arch Clin Exp Ophthalmol.

[29] Plange N, Kaup M, Weber A, Remky A, Arend O (2004). Fluorescein filling defects and quantitative morphologic analysis of the optic nerve head in glaucoma. Arch Ophthalmol.

[30] Plange N, Kaup M, Huber K, Remky A, Arend O (2006). Fluorescein filling defects of the optic nerve head in normal tension glaucoma, primary open-angle glaucoma, ocular hypertension and healthy controls. Ophthalmic Physiol Opt.

[31] Sihota R, Saxena R, Taneja N, Venkatesh P, Sinha A (2006). Topography and fluorescein angiography of the optic nerve head in primary open-angle and chronic primary angle closure glaucoma. Optom Vis Sci.

[32] Talusan ED, Schwartz B (1981). Fluorescein angiography - demonstration of flow pattern of anterior ciliary arteries. Arch Ophthalmol.

[33] Aizawa N, Kunikata H, Yokoyama Y, Nakazawa T (2013). Correlation between optic disc microcirculation in glaucoma measured with laser speckle flowgraphy and fluorescein angiography, and the correlation with mean deviation. Clin Exp Ophthalmol.

[34] Hamard P, Hamard H, Dufaux J, Quesnot S (1994). Optic nerve head blood flow using a laser Doppler velocimeter and haemorheology in primary open angle glaucoma and normal pressure glaucoma. Br J Ophthalmol.

[35] Michelson G, Langhans MJ, Groh MJ (1996). Perfusion of the juxtapapillary retina and the neuroretinal rim area in primary open angle glaucoma. J Glaucoma.

[36] Hafez AS, Bizzarro RL, Lesk MR (2003). Evaluation of optic nerve head and peripapillary retinal blood flow in glaucoma patients, ocular hypertensives, and normal subjects. Am J Ophthalmol.

[37] Sugiyama T, Araie M, Riva CE, Schmetterer L, Orgul S (2010). Use of laser speckle flowgraphy in ocular blood flow research. Acta Ophthalmol.

[38] Konishi N, Tokimoto Y, Kohra K, Fujii H (2002). New laser speckle flowgraphy system using CCD camera. Opt Rev.

[39] Aizawa N, Yokoyama Y, Chiba N, Omodaka K, Yasuda M, Otomo T, Nakamura M, Fuse N, Nakazawa T (2011). Reproducibility of retinal circulation measurements obtained using laser speckle flowgraphy-NAVI in patients with glaucoma. Clin Ophthalmol.

[40] Shiga Y, Omodaka K, Kunikata H, Ryu M, Yokoyama Y, Tsuda S, Asano T, Maekawa S, Maruyama K, Nakazawa T: **Waveform analysis of ocular blood flow and the early detection of normal-tension glaucoma.** *Invest Ophthalmol Vis Sci* In press 10.1167/iovs.13-12930 24130177

[41] Takahashi H, Sugiyama T, Tokushige H, Maeno T, Nakazawa T, Ikeda T, Araie M (2013). Comparison of CCD-equipped laser speckle flowgraphy with hydrogen gas clearance method in the measurement of optic nerve head microcirculation in rabbits. Exp Eye Res.

[42] Wang L, Cull GA, Piper C, Burgoyne CF, Fortune B (2012). Anterior and posterior optic nerve head blood flow in nonhuman primate experimental glaucoma model measured by laser speckle imaging technique and microsphere method. Invest Ophthalmol Vis Sci.

[43] Kim NR, Lee ES, Seong GJ, Kim JH, An HG, Kim CY (2010). Structure-function relationship and diagnostic value of macular ganglion cell complex measurement using Fourier-domain OCT in glaucoma. Invest Ophthalmol Vis Sci.

[44] Seong M, Sung KR, Choi EH, Kang SY, Cho JW, Um TW, Kim YJ, Park SB, Hong HE, Kook MS (2010). Macular and peripapillary retinal nerve fiber layer measurements by spectral domain optical coherence tomography in normal-tension glaucoma. Invest Ophthalmol Vis Sci.

[45] Kim NR, Lee ES, Seong GJ, Kang SY, Kim JH, Hong S, Kim CY (2011). Comparing the ganglion cell complex and retinal nerve fibre layer measurements by Fourier domain OCT to detect glaucoma in high myopia. Br J Ophthalmol.

[46] Tamaki Y, Nagahara M, Araie M, Tomita K, Sandoh S, Tomidokoro A (2001). Topical latanoprost and optic nerve head and retinal circulation in humans. J Ocul Pharmacol Ther.

[47] Tsuda S, Yokoyama Y, Chiba N, Aizawa N, Shiga Y, Yasuda M, Yokokura S, Otomo T, Fuse N, Nakazawa T (2013). Effect of topical tafluprost on optic nerve head blood flow in patients with myopic disc type. J Glaucoma.

[48] Tamaki Y, Araie M, Muta K (1999). Effect of topical dorzolamide on tissue circulation in the rabbit optic nerve head. Jpn J Ophthalmol.

[49] Ishii K, Araie M (2000). Effect of topical timolol on optic nerve head circulation in the cynomolgus monkey. Jpn J Ophthalmol.

[50] Quaranta L, Miglior S, Floriani I, Pizzolante T, Konstas AG (2008). Effects of the timolol-dorzolamide fixed combination and latanoprost on circadian diastolic ocular perfusion pressure in glaucoma. Invest Ophthalmol Vis Sci.

[51] Quaranta L, Katsanos A, Russo A, Riva I (2013). 24-hour intraocular pressure and ocular perfusion pressure in glaucoma. Surv Ophthalmol.

[52] Quaranta L, Gandolfo F, Turano R, Rovida F, Pizzolante T, Musig A, Gandolfo E (2006). Effects of topical hypotensive drugs on circadian IOP, blood pressure, and calculated diastolic ocular perfusion pressure in patients with glaucoma. Invest Ophthalmol Vis Sci.

[CR53] The pre-publication history for this paper can be accessed here: http://www.biomedcentral.com/1471-2415/14/113/prepub

